# The role of Sfrp and DKK proteins in cardiomyocyte development

**DOI:** 10.14814/phy2.14678

**Published:** 2021-02-15

**Authors:** Ying‐Chang Hsueh, Conrad P. Hodgkinson, Jose A. Gomez

**Affiliations:** ^1^ Mandel Center for Heart and Vascular Research, and the Duke Cardiovascular Research Center Duke University Medical Center Durham NC USA; ^2^ Department of Medicine Clinical Pharmacology Division Vanderbilt University Medical Center Nashville TN USA

**Keywords:** cardiomyocyte, DKK, Sfrp2

## Abstract

In this review, we summarize the role of Wnt proteins in cardiomyogenesis. More specifically, we focus on how the development of cardiomyocytes from precursor cells involves a complex interplay between Wnt canonical β‐catenin signaling pathways and Wnt noncanonical signaling pathways involving PCP and JNK. We also describe recent literature which suggests that endogenous Wnt inhibitors such as the Sfrp and DKK proteins play important roles in regulating the cardiomyocyte differentiation.

## Graphical abstract

Srfp2 mediates cardiomyocyte differentiation by inhibiting Wnt3a. Wnt3a is a canonical Wnt that activates b‐catenin and inhibits cardiac specification. In contrast, Wnt11 is a noncanonical Wnt, inhibits b‐catenin and promotes differentiation into cardiomyocytes. The addition of Sfrp2 upsets this balance as it binds, and sequesters, Wnt3a but not Wnt11; thereby leaving Wnt11 free to bind to Fzd5 and activate the noncanonical pathway resulting in cardiac differentiation.

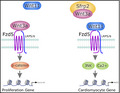

## 1 INTRODUCTION

Cardiomyocytes are specialized muscle cells found solely in the heart. Their main function is to provide the contractile force necessary for the heart to pump blood. In mammals, cardiomyocytes appear early in the developing fetus where they develop from undifferentiated precursors. In this review, we will discuss the importance of the Wnt signaling pathway for cardiomyocyte differentiation.

## 2 WNT PATHWAY OVERVIEW

**Figure 1:**
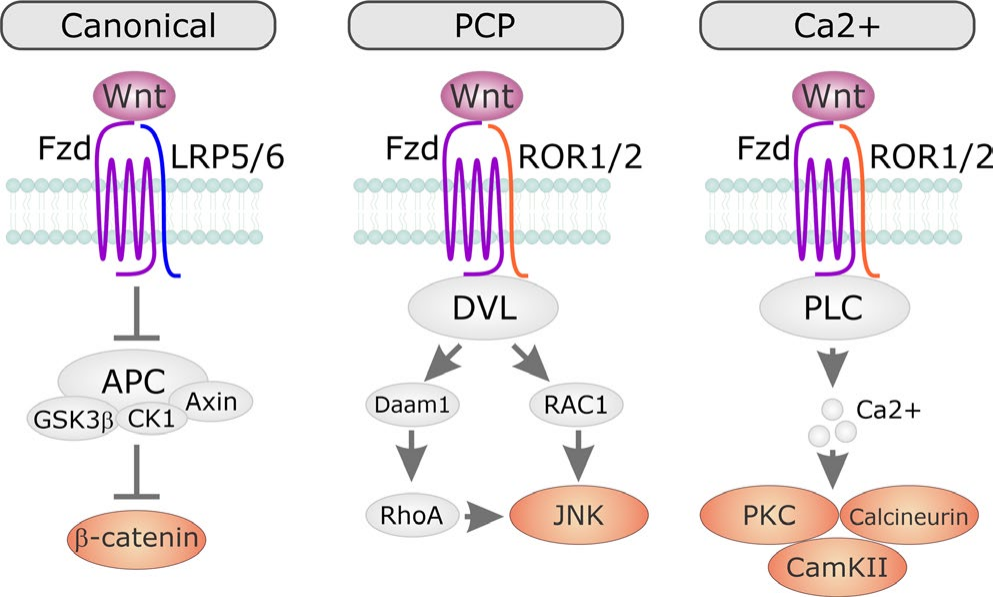
Wnt signaling pathway. Wnt proteins signal via three pathways. The first pathway is known as the canonical β‐catenin pathway. There are also two noncanonical Wnt signaling pathways; the planar cell polarity (PCP) pathway and the Ca^2^+ pathway

Currently, 19 Wnt proteins have been identified in humans and rodents (Gao & Chen, 2010; Giles et al., 2003; Kikuchi et al., 2011). Wnt proteins are secreted from the cell where they act as paracrine or autocrine factors; influencing target cell behavior by binding to Frizzled (Fzd) receptors in the plasma membrane (Foulquier et al., 2018). Porcupine (PORNC), a membrane‐associated O‐acyl transferase enzyme in endoplasmic reticulum (ER), modifies Wnt proteins by palmitoylation (Kadowaki et al., (1996); Lorenowicz & Korswagen, 2009). Palmitoylation is a critical process for Wnt secretion and downstream signaling (Willert & Nusse, 2012). Following secretion, Wnt proteins bind to Fzd receptors; a family of G protein‐coupled receptors (Zhang et al., 2018) which activate β‐catenin dependent and independent signaling pathways (Dawson et al., 2013; Yin et al., 2018). Wnt signaling also requires the lipoprotein‐related receptor (LRP) 5 and 6. LRP5 and LRP6 stabilize the Wnt/Fzd complex at the cell surface (Niehrs & Shen, 2010). Wnt proteins either activate a canonical signaling pathway involving β‐catenin or noncanonical pathways involving planar cell polarity (PCP) or protein kinase C (PKC) (Figure 1).

## 3 OVERVIEW OF WNT SIGNALING PATHWAYS

Following binding to Wnt, the Fzd receptor will activate either a β‐catenin dependent (canonical) or β‐catenin independent (non‐canonical) signaling pathway.

### 3.1 The Wnt/β‐catenin‐dependent pathway

The β‐catenin‐dependent pathway is regulated by a cytoplasmic complex comprised of Axin, glycogen synthase kinase 3β (GSK3 β), adenomatous polyposis coli (APC), and casein kinase 1α (CK1α). The role of this complex is to phosphorylate β‐catenin. Following phosphorylation, β‐catenin associates with E3‐ubiquitin and is degraded (Dawson et al., 2013). When Wnt binds to Fzd, the activated Fzd binds to the Axin/GSK3 β/APC/CK1α complex. This sequesters the complex at the plasma membrane where it is no longer able to phosphorylate β‐catenin. This leads to β‐catenin accumulation in the cytoplasm (MacDonald et al., 2009) and subsequent translocation to the nucleus where it activates gene transcription (MacDonald et al., 2009) via interactions with the TCF/LEF family of proteins (Cadigan & Waterman, 2012). While the TCF/LEF proteins have DNA‐binding ability but require the transactivation domain of β‐catenin to regulate transcription (Cadigan & Waterman, 2012).

### 3.2 The Wnt/β‐catenin independent pathway

A number of Wnts do no activate β‐catenin. Instead, when they bind to Fzd they activate what are commonly called noncanonical or β‐catenin independent signaling pathways. There are two non‐canonical Wnt signaling pathways. The first is the planar cell polarity (PCP) pathway (Adler, 2012). In the PCP pathway, Fzd activates the kinase c‐Jun N‐terminal kinase (JNK). Activated JNK regulates asymmetric cytoskeletal organization and cell polarization (Yang & Mlodzik, 2015). The second non‐canonical pathway is the Wnt/Ca^2+^ pathway. Here, Fzd binding promotes the release of intracellular Ca^2+^. Increased intracellular Ca^2+^ activates phospholipase C (PLC) and protein kinase C (PKC) (Cook et al., 1996). Moreover the phosphatase calcineurin is also activated; leading to dephosphorylation of the transcription factor nuclear factor of activated T‐cells (NFAT) and its accumulation in the nucleus (Kohn & Moon, 2005). Importantly, both noncanonical pathways inhibit β‐catenin (Ackers & Malgor, 2018; Bisson et al., 2015).

## 4 WNT SIGNALING IN HEART DEVELOPMENT

Early expression in the developing heart of canonical Wnts (Wnt2, Wnt2b) and non‐canonical Wnts (Wnt8a, Wnt11) suggests that both the β‐catenin‐dependent and β‐catenin independent signaling pathways are necessary for normal heart development (Tian et al., 2010a).

**Figure 2:**
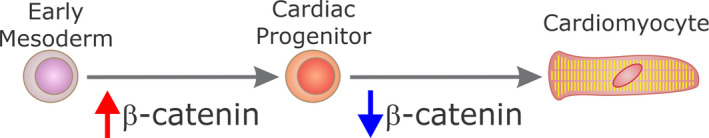
Role of β‐catenin in cardiomyocyte differentiation. Activation of β ‐catenin in mesoderm cells is necessary to generate cardiac progenitors. Subsequent differentiation of these cardiac progenitors into cardiomyocytes requires β‐catenin inhibition

Activation of the Wnt/β‐catenin‐dependent pathway plays a critical role in the formation and subsequent expansion of cardiac progenitor cells in the mesoderm (Huelsken et al., 2000) (Figure 2). Reduced β‐catenin expression prevents the formation of the SHF; decreased cell number; as well as the development of right ventricle and outflow tract (Ai et al., 2007; Klaus et al., 2007). The initial formation appears to be regulated by Wnt1 and Wnt3a; two canonical Wnts that activate β‐catenin. While Wnt1 regulates outflow track and cardiac neural crest development (Brault et al., 2001); Wnt3a is necessary for mesoderm formation (Liu et al., 1999). Prior to differentiation, cardiac progenitors within the mesoderm undergo a period of proliferation. The period of cardiac progenitor proliferation is known to be dependent upon Wnt2; a Wnt which activates β‐catenin (Buckingham et al., 2005; Norden et al., 2011; Tian et al., 2010b). The importance of Wnt2 in cardiomyocyte development has been further demonstrated in vitro. Cardiac progenitors derived from embryonic bodies prepared from Wnt2 knockout mice, proliferate poorly and show limited differentiation into cardiomyocytes (Wang et al., 2007). The SWI/SNF component BAF250a appears to be necessary to direct b‐catenin to the promoters of proliferation genes (Lei et al., 2019).

The duration of Wnt/β‐catenin signaling appears to be important for the subsequent fate of the cardiac progenitors. Modeling in iPS cells indicates that prolonged activation of b‐catenin induces cardiac progenitors to develop into cardiac fibroblasts (Zhang et al., 2019). In contrast, in a subset of cardiac progenitors the initial activation of canonical Wnt/β‐catenin signaling is relatively short‐lived as a feedback loop activates the Wnt/β‐catenin‐independent pathway which in turn represses canonical Wnt/β‐catenin signaling (Cohen et al., 2008). In these cardiac progenitors, activation of the Wnt/β‐catenin‐independent pathway induces differentiation into cardiomyocytes (Gessert & Kuhl, 2010). Repression of the Wnt/β‐catenin signaling pathway may involve miR‐184. Studies with differentiating ES cells indicated that Wnt3, the canonical Wnt needed for cardiac progenitor formation, was down‐regulated by miR‐184 during cardiomyocyte differentiation (Liu et al., 2020). (Gessert & Kuhl, 2010) Activation of the Wnt/β‐catenin‐independent pathway appears to be controlled by Wnt5 and Wnt11 (Cohen et al., 2012). Modeling of heart development in the culture dish has shown that Wnt11 administration induces cardiac progenitors derived from human (Ardehali et al., 2013) and mouse (Pandur et al., 2002) embryonic stem cells to differentiate into cardiomyocytes in vitro. Similarly, Wnt5a induces hemangioblasts to differentiate to cardiomyocytes(Chen et al., 2008). Interestingly, Wnt5 and Wnt11 promote cardiomyocyte differentiation via alternative signaling pathways. While Wnt5 promotes cardiomyocyte differentiation via the Notch pathway (Chen et al., 2008); Wnt11 regulates cardiomyocyte differentiation via PKC and Jun amino‐terminal kinase (JNK) signaling pathways (He et al., 2011).

While the evidence provided so far indicates that cardiomyocyte differentiation requires an initial burst of β‐catenin activation followed by β‐catenin inhibition (Gessert & Kuhl, 2010; Lian et al., 2013) (Figure 2); the finding that continuous b‐catenin activation promotes cardiac progenitor differentiation into fibroblasts suggests that further mechanisms must exist to direct subsets of cardiac progenitors to a particular cell fate. Addressing this question is particularly pertinent considering that the temporal expression patterns of Wnts that activate β‐catenin and β‐catenin‐independent signaling pathways are similar (Tian et al., 2010a). Such research is in its infancy; however, possibilities include spatial position of the cardiac progenitors and differences in extracellular matrix composition. With respect to spatial positioning, canonical b‐catenin signaling via Wnt5b promotes cardiac progenitors to differentiate into cardiac pacemaker cells only if the cardiac progenitors are in outlying mesoderm (Ren et al., 2019). Similarly, extracellular matrix can direct cardiac progenitors to different cell fates (Ding et al., 2020; Hodgkinson et al., 2014).

## 5 WNT SIGNALING IN HEART INJURY AND DISEASE

Cardiac regeneration differs greatly among species. Lower vertebrates such as amphibians and some fish can fully regenerate their heart following injury (Ozhan & Weidinger, 2015). Activation of Wnt/β‐catenin signaling is observed; however, cardiac regeneration is believed to involve cardiomyocyte replenishment following a period of proliferation in a de‐differentiated state (Ozhan & Weidinger, 2015). Higher vertebrates, including all mammals, are unable to regenerate their hearts following injury. The possible role of manipulating Wnt pathways to stimulate cardiomyocyte differentiation via undifferentiated precursors has been sidelined by the controversies surrounding the existence of cardiac progenitors in vivo (He et al., 2020).

Following cardiac injury in mammals the heart undergoes maladaptive remodeling. Commensurate with this remodeling, Wnt/β‐catenin pathways are activated (Hermans & Blankesteijn, 2015). Wnts that activate β‐catenin appear to play a number of roles that impact maladaptive remodeling. Fibrosis, which impairs cardiac function, requires Wnt1 stimulation of cardiac fibroblast proliferation and differentiation (Deb, 2014). Vascularization of the scar tissue is regulated by Wnt1, Wnt3, and Wnt5a (Deb, 2014).

Conversely, inhibition of Wnt/β‐catenin signaling appears to reduce maladaptive remodeling. For example, inactivation of b‐catenin specifically in cardiac fibroblasts reduces fibrosis (Xiang et al., 2017). Similarly, knockout of disheveled (Dvl) protein, a component of the β‐catenin signaling pathway, attenuated pressure‐overload induced cardiac hypertrophy (Schans et al., 2007).

Macrophages play important anti‐ and pro‐regenerative functions in the post‐injury heart (Hodgkinson et al., 2016). The balance between the anti‐ and pro‐regenerative functions may be dependent upon Wnts. For example, Wnt5a stimulates fibroblasts to secrete pro‐inflammatory cytokines such IL6 (Abraityte et al., 2017) and IL6 induces macrophages to develop into an (Hodgkinson et al., 2016) anti‐regenerative phenotype (Hodgkinson et al., 2016). Conversely, ablation of the b‐catenin pathway induces macrophages to switch to a pro‐regenerative phenotype (Palevski et al., 2017).

## 6 SFRPS AND DKKS: ENDOGENOUS WNT INHIBITORS

Wnt proteins are regulated by other secreted molecules. One important class of these secreted molecules are Wnt inhibitors. These Wnt inhibitors are grouped into two families: Secreted frizzled‐related proteins (Sfrps) and Dickkopf (DKK) proteins. Both Sfrp and DKK proteins play important roles in cardiac differentiation.

### 6.1 Secreted frizzled‐related proteins (Sfrp)

**Figure 3:**
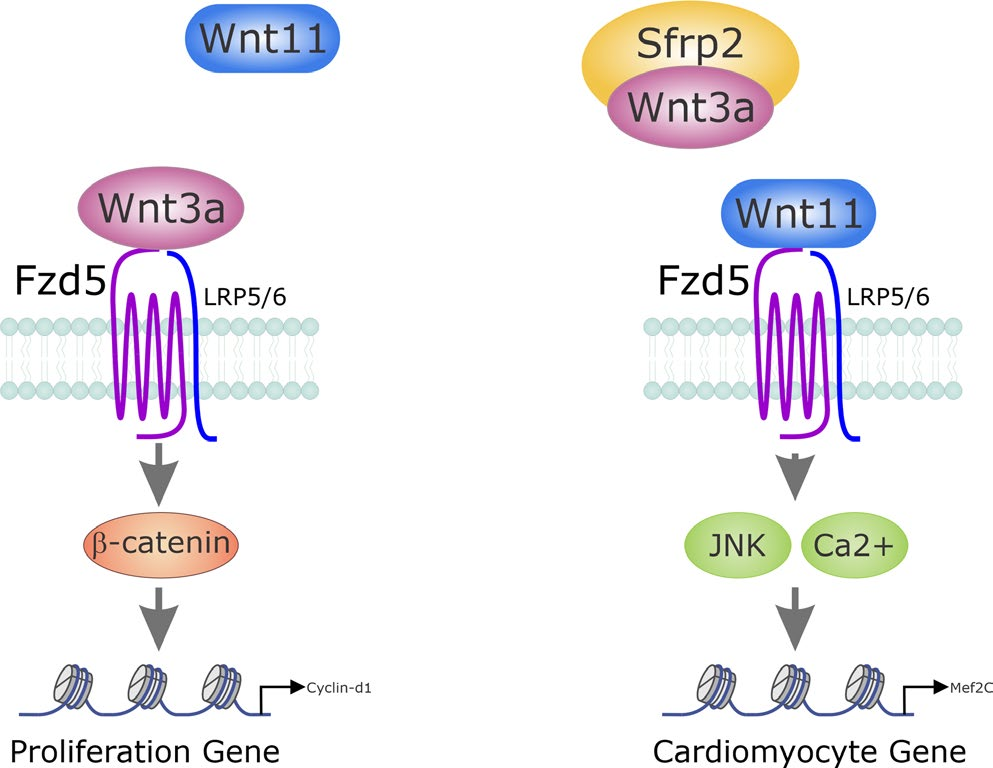
Srfp2 mediates cardiomyocyte differentiation by inhibiting Wnt3a. Undifferentiated cells express both Wnt3a and Wnt11. Wnt3a is a canonical Wnt that activates β‐catenin and inhibits cardiac specification. In contrast, Wnt11 is a noncanonical Wnt, inhibits β‐catenin and promotes differentiation into cardiomyocytes. The competing roles of Wnt3 and Wnt11 prevent cardiomyocyte differentiation. The addition of Sfrp2 upsets this balance as it binds, and sequesters, Wnt3a but not Wnt11; thereby leaving Wnt11 free to bind to Fzd5 and activate the non‐canonical pathway resulting in cardiac differentiation

Humans have five Sfrp (Sfrp1‐5). These Sfrps have cysteine‐rich (CRD) domains with 10 conserved Cys residues. As their name suggests, Sfrps have a strong (~30% to 50%) homology with Fzd receptors and their function is to compete with Fzd receptors for Wnt binding. In essence, Sfrps act as Wnt inhibitors (Chong et al., 2002). With respect to cardiomyocyte development, Sfrp1 and Sfrp4 inhibit Wnt3a; the Wnt necessary for cardiac progenitor development (Bovolenta et al., 2008; Wawrzak et al., 2007). However, Sfrps appear to enhance cardiac progenitor differentiation. This appears to be due to enhancing noncanonical Wnt signaling. For example, Sfrp1 enhances Wnt5a stimulation of cardiomyocyte differentiation from embryonic stem cells (Chen et al., 2008). Similarly, Sfrps also enhance noncanonical signaling by directly inhibiting canonical Wnts. Sfrp1 improved cardiac stem cell differentiation in *Xenopus* by inhibiting the canonical Wnt6 pathway (Gibb et al., 2013). In addition, we have also shown recently that Sfrp2 binding to Wnt6 promotes Sca‐1+ cells to express cardiomyocyte‐specific proteins (Schmeckpeper et al., 2015). In this study, we discovered that Wnt6 prevents cardiomyocyte differentiation via activation of β‐catenin (Schmeckpeper et al., 2015). Sfrp2 binding Wnt6 induced the activation of Wnt non‐canonical PCP and JNK signaling pathways. These noncanonical Wnt signaling pathways were found to promote cardiomyocyte‐specific gene expression in undifferentiated cells via β‐catenin inhibition (Schmeckpeper et al., 2015). To further understand the role of Sfrp2 in cardiomyocyte differentiation we performed an RNA‐seq comparing undifferentiated cells, undifferentiated cells incubated with Sfrp2 and cardiomyocytes. Importantly, these cells were freshly isolated from the heart to avoid any artefacts arising from culturing. We discovered a central for Fzd5. Undifferentiated cells express both Wnt3a and Wnt11. Wnt3a is a canonical Wnt that activates β‐catenin and inhibits cardiac specification (Cohen et al., 2008; Oikonomopoulos et al., 2011; Yamashita et al., 2005). In contrast, Wnt11 is a non‐canonical Wnt, inhibits β‐catenin and promotes ES cell differentiation into cardiomyocytes (Mazzotta et al., 2016). Consequently, we proposed a mechanism whereby undifferentiated cells do not normally develop into cardiomyocytes due to competing roles of Wnt3a and Wnt11. The addition of Sfrp2 upsets this balance as it binds, and sequesters, Wnt3a but not Wnt11; thereby leaving Wnt11 free to bind to Fzd5 and activate the non‐canonical pathway resulting in β‐catenin inhibition and cardiac differentiation (Hodgkinson et al., 2018) (Figure 3).

### 6.2 Dickkopf (DKK)

DKK proteins inhibit Wnt signaling by binding to LRP5 and LRP6 (Joiner et al., 2013). This prevents LRP5 and LRP6 from stabilizing the Wnt/Fzd complex at the plasma membrane (Dawson et al., 2013). DKK proteins, like Sfrps, appear to enhance cardiac progenitor differentiation. DKK1 administered to Flk1+ cells derived embryonic stem cells improves their ability to differentiate. Interestingly, the addition of DKK1 before cardiac progenitor cell formation prevents the appearance of cardiomyocytes; mimicking the role of β‐catenin inhibition and activation in heart development (Yamashita et al., 2005; Yang et al., 2008).

### 6.3 The role of endogenous Wnt inhibitors in cardiac development

Cardiac development, as described above, requires biphasic regulation of the Wnt/β‐catenin pathway. In the first instance, activation of the Wnt/β‐catenin pathway is necessary to produce cardiac progenitors. Subsequent differentiation of these cardiac progenitors requires Wnt/β‐catenin pathway inhibition. Endogenous Wnt inhibitors such as Sfrp and DKK proteins can play both positive and negative roles on cardiac development depending upon their temporal and spatial pattern of expression.

Sfrp1, Sfrp2, and Sfrp5 are closely related and appear to play similar roles in cardiac development as well as maintenance of the heart. Expression of these Sfrps is found in the mesoderm and ectoderm of chick embryos (Terry et al., 2000); as well as in the developing mouse heart (Satoh et al., 2008). In vitro experiments suggest that Sfrp2 may inhibit the specialization of mesoderm cells into cardiac progenitors (Deb et al., 2008). However, in vivo experiments support the notion that Sfrp2, as well as Sfrp1 and Sfrp5, promote cardiac development. Sfrp1, Sfrp2, and Sfrp5, were shown to be necessary for somitogenesis (Satoh et al., 2008). Interestingly, Sfrp5 also marks cardiac progenitors that are destined to become the outflow tract, left ventricle, atrium, and sinus venosus (Fujii et al., 2017). Considering that the differentiation of cardiac progenitors into cardiomyocytes requires Wnt/β‐catenin inhibition, expression of Sfrp5 in cardiac progenitors suggests that an autocrine loop maybe involved in their subsequent differentiation. Further evidence for a role in cardiac development comes from experiments where Sfrp proteins were injected into the injured heart. Here, Sfrp2 was found to induce undifferentiated cells to express cardiomyocyte‐specific genes and proteins (Hodgkinson et al., 2018; Schmeckpeper et al., 2015),. With respect to signaling mechanisms, Sfrp proteins act partly through inhibition of Wnt/β‐catenin signaling. However, the Sfrp proteins also utilize noncanonical Wnt signaling pathways such as the Planar Cell Polarity and JNK pathways (Hodgkinson et al., 2018; Satoh et al., 2008; Schmeckpeper et al., 2015). Beyond regulation of cardiomyocyte development, it also appears that continued Sfrp expression is needed to maintain the heart. Deletion of the Sfrp1 gene deletion leads to abnormal cardiac structure that worsens with age (Sklepkiewicz et al., 2015). Moreover these changes in cardiac structure impair cardiac function (Sklepkiewicz et al., 2015).

Akin to Sfrps, the DKK family also play important roles in cardiac development. Loss of function approaches have shown that DKK1 is necessary for cardiomyocyte formation in Xenopus laevis (Guo et al., 2019) and heart development in the chicken embryo (Marvin et al., 2001). While DKK1 is necessary for cardiomyocyte formation, it apparently plays no further role in the specification of cardiomyocytes into their ventricular, aortic, or pace‐maker subtypes (Guo et al., 2019). DKK1 regulates Xenopus laevis axis formation via a Wnt5/Wnt11 complex, inducing a change in canonical β‐catenin signaling to non‐canonical JNK (Cha et al., 2008), and could potentially act in a similar fashion in cardiomyocyte differentiation. At the transcriptional level, DKK1 may regulate gene transcription via the HEX transcription factor as HEK loss‐of‐function experiments prevent DKK1 from inducing endogenous heart development and ectopic heart induction (Foley & Mercola, 2005). While DKK2 and DKK3 are expressed in the developing heart (Monaghan et al., 1999), little is known of their roles in cardiac development.

### 6.4 Endogenous Wnt inhibitors in cardiac injury, repair and regeneration

Following cardiac injury, fibrosis becomes a significant issue. Fibroblasts proliferate and deposit extracellular matrix proteins. The deposition of extracellular matrix proteins prevents the heart from functioning normally; resulting in heart failure and arrhythmia. Endogenous Wnt inhibitors, notably Sfrps, play important roles in the fibrotic response. In general, the available data suggests that Sfrp1 inhibits fibrosis. Genetic ablation of the Sfrp1 gene increases the expression of several Wnts, β‐catenin, as well as the Wnt target genes Lef1 and Wisp1. Increased Wisp1 expression promotes fibrosis by inducing fibroblasts to proliferate and produce fibroblast production of α‐smooth muscle and collagen (Konigshoff et al., 2009; Sklepkiewicz et al., 2015).

In contrast to Sfrp1, the role of Sfrp2 in fibrosis is unclear. Sfrp2 expression is increased during fibrosis and genetic ablation of Sfrp2 reduces collagen deposition (Kobayashi et al., 2009). Similarly, the injection of a Sfrp2 antibody into the failing hamster heart reduced myocardial fibrosis (Schumann et al., 2000). In further support of a role of Sfrp2 in promoting fibrosis, Sfrp2 induces tissue non‐specific alkaline phosphatase which acts on tolloid‐like metalloproteinases to promote collagen maturation (Martin et al., 2015). In contrast to these two studies, injection of Sfrp2 into the infarcted rat heart had the opposite effect and reduced fibrosis (He et al., 2010). In this study, Sfrp2 was found to inhibit fibrosis by inhibiting BMP4 mediated processing of collagen (He et al., 2010). The disparity between these studies may be due to the dose of Sfrp2 employed as high doses of Sfrp2 inhibit fibrosis, whereas low doses promote fibrosis (Mastri et al., 2014).

Beyond a direct role in mediating the damaging effects of cardiac injury, Sfrps have also attracted much interest as cardio‐protective agents. The effects of Sfrp1 on cardiomyocyte apoptosis appear to be context specific. In an ischemic pre‐conditioning model of cardiac injury, Sfrp1 over‐expression increased cardiomyocyte apoptosis and increased infarct size (Barandon et al., 2005) via activation of GSK‐3β. However, in a coronary artery ligation injury model, Sfrp1 over‐expression had the opposite effect; reducing cardiomyocyte apoptosis and correspondingly reducing the size of the infarct (Barandon et al., 2003). Similarly, in a transverse aortic constriction (TAC)‐induced model of heart failure, Sfrp1 attenuated cardiac dysfunction by inhibiting cardiomyocyte apoptosis (Pan et al., 2018). In light of these divergent results, Hu and colleagues recently suggested that the effects of Sfrp1 on cardiomyocyte apoptosis are location dependent (Hu et al., 2019). The authors of this study found that extracellular Sfrp1 enhanced Doxycycline‐induced cardiotoxicity by suppressing Wnt/β‐catenin signaling; whereas Sfrp1 in the intracellular compartment of cardiomyocytes protected against Doxycycline‐induced cardiomyocyte apoptosis by interacting with PARP1 (Hu et al., 2019). Sfrp2 has also been shown to regulate cardiomyocyte apoptosis. Both in vitro and in vivo, Sfrp2 reduced cardiomyocyte apoptosis by binding to Wnt3a and decreasing caspase activity (Zhang et al., 2009). Similar to the effects on cardiomyocyte differentiation, the effects of Sfrp2 on cardiomyocyte apoptosis via Wnt3a sequestration may involve non‐canonical Wnt signaling pathways. For example, Sfrp2 reduces UV‐induced apoptosis in primary cultures of canine mammary gland tumors via the activation of NF‐kappaB and inhibition of JNK (Lee et al., 2006). Similar to Sfrp2, DKK3 inhibits also cardiomyocyte apoptosis via JNK inhibition (Cao et al., 2018; Zhang et al., 2014). Interestingly, expression of Sfrp3 and Sfrp4 positively correlates with the expression of apoptosis related genes in the failing human heart (Schumann et al., 2000). A more direct role for Sfrp4 in regulating cardiomyocyte apoptosis was shown in a Sfrp4 knockdown study involving ischemia‐reperfusion cardiac injury. Here, loss of Sfrp4 expression reduced injury by preventing the expression of pro‐apoptotic Bax, caspase‐3, and Bcl‐2 genes in cardiomyocytes (Zeng et al., 2019). It is currently unknown if Sfrp3 and Sfrp4 promote cardiomyocyte apoptosis via Wnt dependent pathways.

### 6.5 Signaling crosstalk

While Sfrps and DKKs are typically thought of in their role as Wnt inhibitors, it is important to note that the Wnt signaling pathway itself shows significant crosstalk with other cellular pathways including the Notch, ROS and NF‐kappaB pathways. The interactions between these pathways are complex and the reader is referred to many excellent reviews on the subject (Caliceti et al., 2014; He et al., 2006; Ma & Hottiger, 2016).

## 7 SUMMARY

Wnt signaling pathways regulate cardiomyocyte differentiation by β‐catenin dependent (canonical) and β‐ctenin independent (non‐canonical) regulation. Wnt/β‐catenin activation in the mesodermal stage promotes the formation of progenitor cells and their differentiation. Critical roles have been ascribed to LRP5/6 and Wnt3a. Inhibition of β‐catenin by Wnt non‐canonical pathways is then necessary for the cardiac progenitors to differentiate into cardiomyocytes. Important roles have been ascribed to Wnt5a and Wnt11. Importantly, Wnt inhibitors such as DKK1, Sfrp1, and Sfrp2 also play important roles in switching signaling from β‐catenin activation to β‐catenin inhibition. This bi‐phasic switch in β‐catenin signaling has already found use in differentiating of induced pluripotent stem cells (iPSCs) into cardiomyocytes (Burridge et al., 2014; Lian et al., 2013). While the research is still in its’ infancy, spatial and temporal treatment of the mammalian heart with β‐catenin inhibitors such as DKK1, Sfrp1, and Sfrp2 may play critical roles in improving heart function after injury by inducing undifferentiated precursors to differentiate into new cardiomyocytes.

## CONFLICT OF INTEREST

No conflicts of interest, financial or otherwise, are declared by the authors.

## AUTHOR CONTRIBUTIONS

Y‐C.H. wrote the manuscript, figures, and table draft. C.P.H. and J.A.G. edited the paper, figures and table, and approved the final version.

## ETHICAL STATEMENT

Dr. Gomez is an Assistant Professor at Vanderbilt University Medical Center. Dr. Gomez laboratory is funded by an NHLBI Research Scientist Development Grant (1K01HL135461), and in part by discretionary research funds from the Vanderbilt University Medical Center.
